# CONTEXTUAL FACTORS IN AGING AND LONG-TERM SERVICES AND SUPPORTS RESEARCH

**DOI:** 10.1093/geroni/igad104.2035

**Published:** 2023-12-21

**Authors:** Chanee Fabius, Katherine Ornstein

## Abstract

In the coming years, there will be more older Americans living in the community with extensive medical and long-term care needs increasing the need for long-term services and supports (LTSS) that assist with routine daily activities (e.g., bathing, dressing, medication management). Increasingly research studies are examining factors associated with receipt of LTSS including care preference, family availability and financial resources. Yet LTSS care experiences are likely a result of factors that extend beyond personal preference or individual factors, such as neighborhood quality. availability of home care workers, and state policy characteristics (e.g., state Medicaid generosity). Little research to date has examined how the LTSS environmental context affects the health and well-being of older adults with disabilities and their caregivers. In order to improve LTSS research we need to expand analyses to consider relevant contextual factors. This symposium will feature five presentations that provide novel insight regarding social and physical contextual factors contributing to LTSS using multiple data sources. We will describe (1) a conceptual framework of LTSS-relevant environmental domains and associations between select LTSS-relevant characteristics and older adult care experiences, (2) the relationship between neighborhood deprivation and place of death, (3) care experiences of older Medicare-Medicaid enrollees living in counties with Medicaid Managed Long-Term Services and Supports, (4) trends in paid home care employment and state public financing, and (5) associations between adverse consequences due to unmet, LTSS use, and social isolation.

